# Probabilistic Random Forest improves bioactivity predictions close to the classification threshold by taking into account experimental uncertainty

**DOI:** 10.1186/s13321-021-00539-7

**Published:** 2021-08-19

**Authors:** Lewis H. Mervin, Maria-Anna Trapotsi, Avid M. Afzal, Ian P. Barrett, Andreas Bender, Ola Engkvist

**Affiliations:** 1 Molecular AI, Discovery Sciences, R&D, AstraZeneca, Cambridge, UK; 2 Department of Chemistry, Centre for Molecular Informatics, University of Cambridge, Lensfield Road, Cambridge, CB2 1EW UK; 3 Data Sciences & Quantitative Biology, Discovery Sciences, R&D, AstraZeneca, Cambridge, UK; 4 Molecular AI, Discovery Sciences, R&D, AstraZeneca, Gothenburg, Sweden; 5 Department of Computer Science and Engineering, Chalmers University of Technology, Gothenburg, Sweden

**Keywords:** Probabilistic random forest, Target prediction, Uncertainty estimation, Experimental error, Applicability Domain

## Abstract

Measurements of protein–ligand interactions have reproducibility limits due to experimental errors. Any model based on such assays will consequentially have such unavoidable errors influencing their performance which should ideally be factored into modelling and output predictions, such as the actual standard deviation of experimental measurements (σ) or the associated comparability of activity values between the aggregated heterogenous activity units (i.e., K_i_ versus IC_50_ values) during dataset assimilation. However, experimental errors are usually a neglected aspect of model generation. In order to improve upon the current state-of-the-art, we herein present a novel approach toward predicting protein–ligand interactions using a Probabilistic Random Forest (PRF) classifier. The PRF algorithm was applied toward in silico protein target prediction across ~ 550 tasks from ChEMBL and PubChem. Predictions were evaluated by taking into account various scenarios of experimental standard deviations in both training and test sets and performance was assessed using fivefold stratified shuffled splits for validation. The largest benefit in incorporating the experimental deviation in PRF was observed for data points close to the binary threshold boundary, when such information was not considered in any way in the original RF algorithm. For example, in cases when σ ranged between 0.4–0.6 log units and when ideal probability estimates between 0.4–0.6, the PRF outperformed RF with a median absolute error margin of ~ 17%. In comparison, the baseline RF outperformed PRF for cases with high confidence to belong to the active class (far from the binary decision threshold), although the RF models gave errors smaller than the experimental uncertainty, which could indicate that they were *overtrained* and/or *over-confident*. Finally, the PRF models trained with putative inactives decreased the performance compared to PRF models without putative inactives and this could be because putative inactives were not assigned an experimental pXC_50_ value, and therefore they were considered inactives with a low uncertainty (which in practice might not be true). In conclusion, PRF can be useful for target prediction models in particular for data where class boundaries overlap with the measurement uncertainty, and where a substantial part of the training data is located close to the classification threshold.

**Supplementary Information:**

The online version contains supplementary material available at 10.1186/s13321-021-00539-7.

## Introduction

The application of Machine Learning (ML) and Artificial Intelligence (AI) to the drug development process has increased in recent years [1–3], but the majority of research toward small molecule property prediction itself has predominantly focused on improving the reported accuracy of base algorithms, rather than factoring the experimental error into predictions [4]. Currently, uncertainty estimation as a field is gaining traction due to the application of predictive models toward autonomous decision making within the design-make-test-analyse (DMTA) cycle [5, 6]. Various methodologies have been developed and applied in molecular property prediction models to account for the uncertainty in prediction and/or reliability of prediction [7]. The conformal, calibration and Bayesian procedures, shown in Table 1, have historically focused on the behavioural characteristics of the base estimator itself (or variants thereof) after initial data processing, and so provide limited consideration toward the true uncertainty in the underlying biological data used to train the algorithm. In reality, the maximum achievable accuracy of in silico models depends on the quality of the experimental data (i.e. when models approximate experimental error) [8].

**Table 1:** Description of Methodologies, which are used to take into account uncertainty in predictions, and their advantages and disadvantages

Method	Description	Advantage	Disadvantage
Applicability Domain (AD) estimation	Provides an estimate of whether the assumptions of a model are fulfilled for a given input [42–45], e.g., distance to model AD provides a reliability based on whether a query compound is close to model training data	Provides estimates in uncertainty when making predictions for new compounds	Do not commonly take into account the uncertainty related to the underlying data
Conformal Prediction	Produces error bands around the predictions, with the underlying assumption that inputs less similar to model training data should lead to less certain estimates. This is captured using a nonconformity measure, i.e., the nonconformity score for a new query compound is calculated [46–48]	Provides estimates in uncertainty when making predictions for new compounds	Do not commonly take into account the uncertainty related to the underlying data
Probability Calibration	Addresses the question of obtaining accurate likelihoods of predictions based on the distributions of reference observations for a given dataset [36]	There are advantages related to specific calibration methodologies e.g., Isotonic regression methodology makes no assumptions on the curve form. Inductive methods must split data in order to create ‘proper’ calibration splits	Performance depends on the reference observations used Limitations related to specific calibration methodologies: e.g., Isotonic regression methodology requires a large number of calibration points and has a tendency to overfit
Gaussian processes (GP, Bayesian methodology)	Probability distributions over possible functions are used to evaluate confidence intervals and decide based on those if one should refit the prediction in some region of interest [7]	Allow the incorporation of data prior knowledge The uncertainty of a fitted GP increases away from the training data	Gaussian processes can be computationally expensive (because of their non-parametric nature and they need to take into account all the training data each time they make a prediction)

Since experimental error influences dataset generation and performance, it is important to investigate methods capable of accommodating experimental variability during training. This is particularly important for binary classification tasks due to imposing arbitrary cut-off(s) to the activity scale. Such architectures are frequently applied toward biological tasks with poor regression predictivity, as is the case for in silico target prediction approaches, where binding probabilities for orphan compounds are calculated at one or more activity thresholds [9–12]. Structure activity relationship (SAR) landscapes are highly discontinuous (e.g., presence of activity cliffs) and IC_50_/EC_50_/K_i_/K_d_ activities are often heteroscedastic (i.e., the measurement error is unequally distributed across the range of activity values) so regression is not favourable for in silico target prediction. The main caveat of binary classification approaches is that they weight minority cases close to the threshold boundary equivalently in distinguishing between activity classes. For example, pXC_50_ activity values of 5.1 or 4.9 are treated equally important in contributing to the opposing activity (e.g., classification threshold of 5), even though experimental error may not afford such discriminatory accuracy. This is detrimental in practice and therefore it is equally important to evaluate the presence of experimental error in databases and apply methodologies to account for variability in experiments.

One potential option to remove uncertainty near the classification threshold is the removal of edge cases (i.e., classification marginals), for compounds with activity or property values close to the cut-off value used for classification. This however results in the removal of valuable minority class instances (compounds belonging to the active label) and is likely to hinder the predictivity or applicability of models. For this reason, the removal of “edge cases” of highly imbalanced datasets is not common practice within the field [13] and is considered outside the scope of this work.

Firstly, in order to better understand the deviation of activity values across the different protein targets to be modelled, one must first explore the experimental variability of bioactivity data in chemogenomic repositories. One such study of public bioactivity data was performed by Kramer et al. [14] who analyzed the biological activity data deposited in ChEMBL [15] (version 12) for reproducibility (i.e., the experimental uncertainty of independent measurements). The experimental uncertainty was estimated to yield a mean error of 0.44 pK_i_ units, a standard deviation of 0.54 pK_i_ units, and a median error of 0.34 pK_i_ units. The maximum possible squared Pearson correlation coefficient (R^2^) on large data sets was estimated to be 0.81. Further, the heterogenous use of public biochemical IC_50_ data was shown to be problematic, because they are assay specific and comparable only under certain conditions [16]. This phenomenon is particularly relevant for large scale datasets used in target prediction, since it is not feasible to check each data entry manually and it is commonplace to mix available IC_50_ values from public databases even if assay information is not reported. In a similar manner, Kalliokoski et al. [16], analyzed the types of errors, redundancy and variability in ChEMBL. IC_50_ variability was assessed comparing all pairs of independent IC_50_ measurements on identical protein–ligand systems. The standard deviation of pIC_50_ data (equal to 0.68) was only 25% larger than the standard deviation of K_i_ data, suggesting that mixing IC_50_ data from different assays without knowledge of assay conditions adds a moderate amount of noise to the overall data. The standard deviation of public ChEMBL IC_50_ data, as expected, resulted greater than the standard deviation of in-house intra-laboratory/inter-day IC_50_ data, which showed a standard deviation of pIC_50_ values equal to 0.22 and 0.17 for two different drug-target combinations. Augmenting mixed public IC_50_ data by public K_i_ data was not found to deteriorate the quality of the mixed IC_50_ data, if the K_i_ is corrected by an offset. For the ChEMBL database, a K_i_-IC_50_ conversion factor of 2 was suggested.

Another study reported a median discordance (margin between pXC_50_ values) of 0.48 between laboratory measurements for proteins within the same organism, and 0.42 after discriminating between assay type [17]. Further aggregation of bioactivities observed in human and related (orthologue) biological systems (a common practice during data assimilation to increase data quantity [18, 19]), also increased the median standard deviation to 0.51, respectively. Experimental variability is also prevalent for other biological endpoints. One study explored the experimental uncertainty of cytotoxicity data from ChEMBL and calculated that the maximum achievable Pearson correlation coefficient of in-silico models trained on cytotoxicity data from different laboratories ranged between 0.51–0.85, which is considerably different to a 1.0 coefficient corresponding to perfect reproducibility [20]. Experimental error has also been analysed for proprietary datasets, where a recent AstraZeneca study focused on a systematic evaluation of biological assay variability of all biological assays between 2005 and 2014 [21]. The authors found less than a two-fold difference in the average experimental uncertainty, where EC_50_ and IC_50_ measurements tend to have lower standard deviations (with a standard deviation above 0.5), compared to K_d_ and K_i_ measurements. Novartis analysed randomly picked (repeatedly measured) samples of typical assay endpoints over several years, and calculated a standard pIC_50_ deviation of ~ 0.2 log units [16]. Hence, experimental error is also observed within the same laboratories.

Another factor affecting the deviation of results in bioactivity data is the inconsistent mining and preparation of data for structure–activity modelling. For example, Fourches, et al. [22] emphasised the need for standardised chemical data curation strategies (e.g. curation of chemical structures and biological data) that should be followed at the onset of any molecular modelling investigation to avoid discrepancies. Another study highlighted the importance of data selection and extraction, and proposed the combined application of various query parameters available to any user of the ChEMBL database and other selection criteria (such as common compound promiscuity) to harmonize data retrieval [23]. Moreover, discrepancies between bioactivity data in public databases could arise from errors in the data curation and Tiikkainen, et al. [24] raised awareness on the frequencies and types of errors in bioactivity data. Error rates for three large bioactivity databases, namely ChEMBL (version 14), Liceptor (version 2012_03) and WOMBAT (version 2012.01) were calculated. The authors observed that the ligand structures showed the highest probability of being discrepant followed by the protein target, activity value, and finally the activity type. Errors in activity values mainly arose due to unit conversion issues (e.g., micromolar affinities curated as nanomolar) and the activity type (e.g., IC_50_, K_i_, etc.) are usually clearly stated in the source articles. Hence, curation-related errors increase the possibilities of non-systematic error in public bioactivity datasets and consequently increase uncertainty for ligand-target annotations. The possibility of experimental annotation error should also be accounted for during modelling.

Given the above studies, one can expect a large variation in the range of observed standard deviations between experiments, which should be considered when assimilating a training set dependent on the measurement units and method of aggregation across heterogenous assays. However, there are relatively few previous studies that have framed experimental uncertainty as the natural upper limit of the predictive performance possible, closely monitoring when the maximal performance of a model has been reached [25, 26]. For example, an analysis by Brown, Muchmore and Hajduk [25] explored the influence of assay and prediction errors in predictive modelling for drug discovery. The authors calculated the upper performance limit of a model (i.e. correlation between experimental and predicted value), which is likely to be ~ 80%, given a standard deviation of ~ 0.3 and the dataset comprised a potency range of only 2 log units. The authors suggested levels of toleration based on the requirements of a particular model application. For example, an upper limit of five standard deviations in prediction errors was suggested for prioritising compounds for HTS, versus an upper limit of one standard deviation for lead optimisation models to ensure a degree of “discovery productivity”. Another study took into account the uncertainty in bioactivity data in a systematic analysis of the effect of random experimental errors in the predictive ability of QSAR models. The analysis aimed to evaluate the influence of experimental variability in target prediction models by simulating experimental error on 12 Machine Learning algorithms in bioactivity modelling using 12 diverse data sets (15,840 models in total) from ChEMBL (version 19) [27]. Noise was artificially defined (which may not reflect real-world situations, where systematic differences between labs etc. exist) by sampling a Gaussian distribution with zero mean and a variance value (defined as a function of the range of bioactivities considered in each data set). Model performance on the test set was used as a proxy to monitor the relative noise sensitivity of these algorithms as function of the level of noise added to the bioactivities from the training set. Overall, Gradient Boosting Machines (GBMs) showed a low tolerance to noisy bioactivities although its performance was comparable to RF, Support Vector Machines (SVM) and Gaussian Process (GP) for low noise levels. The other algorithms showed comparable noise tolerance and a linear decrease of model performance by increasing the level of noise. Therefore, the presence of error in the training data affected the performance of all the algorithms tested and hence should be taken into consideration.

A different approach to account for experimental uncertainty is to explore methodologies that are able to deal with experimental variability. One such method is the Bayesian developed “sum-of-trees” model (BART) [28], where each tree is constrained by a regularization prior to be a weak learner, and fitting and inference are accomplished via an iterative Bayesian backfitting MCMC algorithm that generates samples from a posterior. Effectively, BART is a nonparametric Bayesian regression approach using dimensionally adaptive random basis elements. Motivated by ensemble methods and boosting algorithms in particular, BART is defined by a statistical model using a prior and a likelihood. This approach enables posterior inference including point or interval estimates of the unknown regression function as well as the marginal effects of potential predictors. Although this algorithm presents an interesting comparison, MCMC is slow to perform on larger datasets (as in the case of the many millions of inactive bioactivities held in repositories such as PubChem [29]). Another, more computationally efficient option is the probabilistic random forest (PRF) [30], which is a modification of the long-established Random Forest (RF) algorithm, which can take into account uncertainties in the measurements (i.e., features) as well as in the assigned classes (i.e., activity labels). It is an algorithm recently released for dealing with noisy astronomical data and the scope of this paper is to use this novel methodology for target prediction [30, 31].

**Fig. 1:**
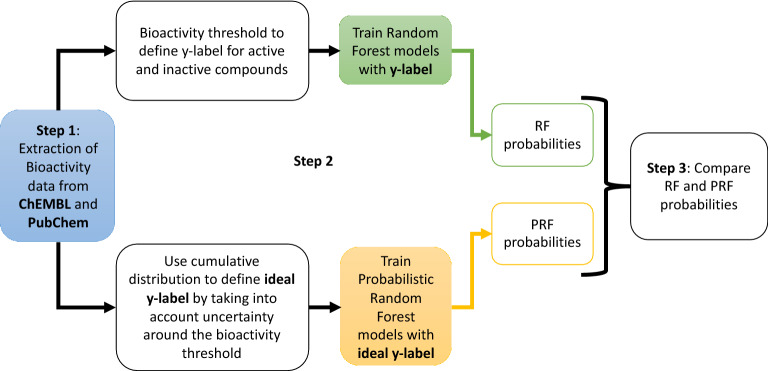
Summary of the analysis performed in this work. Random Forest and Probabilistic Random Forest are used to train models and their output probabilities are compared

We present an approach to utilize the standard deviation of experimental measurements of bioactivity data from the ChEMBL and PubChem repositories, by using the PRF algorithm. The workflow employed in this analysis can be divided into three main steps (see Fig. 1). Step 1 is the extraction of bioactivity data from ChEMBL and PubChem databases. Step 2 is the training of models with two different types of algorithms. The first is the classic Random Forest (RF) and the second a modified version of the original RF, namely the Probabilistic Random Forest (PRF), which is able to take into account uncertainties in assigned classes (i.e., y-labels). The main difference between the two algorithms is that RF uses discrete variables for the activity label (y-label), which is defined by applying a bioactivity threshold in the bioactivity data for each target modelled. On the other hand, PRF algorithm treats the labels as probability distribution functions, rather than deterministic quantities (and we refer to as “ideal y-label”). We train multiple PRF models where we inject different types of noise into bioactivity data. Finally, Step 3 of this work includes the comparison between the probabilities returned from RF and PRF algorithms. With this approach, we present, to our knowledge, for the first time an application of probabilistic modelling of activity data for target prediction using a novel algorithm, which is a modification of the well-established RF algorithm.

## Methods

### Bioactivity data set

The ChEMBL (version 27) database [32] was filtered for compounds with a reported pChEMBL (normalized − log^10^) activity value from ‘binding’ (IC_50_/EC_50_/K_i_/K_d_) human protein assays. Confidence scores of 5 and 8 were employed for the reproducibility comparison when activity values were aggregated at protein complexes or for specific individual proteins, respectively. Compounds were subsequently filtered for a confidence score of 8 for modelling purposes. Targets were also subsequently filtered for greater or equal to 50 active compounds across the activity thresholds for the pChEMBL activity bins 5, 6 and 7 (corresponding to activity values 10, 1 and 0.1 μM) to ensure that only proteins encompassing sufficient chemical space across the activity thresholds were retained for the training set. Models were trained for 559 targets and Additional file 1: Figure S1 summarizes the number of active and inactive data points for each model and for which a large variance between the amount of bioactivity data available per target was observed. For example, there was a median of 389, 375, and 386 active compounds per-target for the pChEMBL classification thresholds of 5, 6 and 7, respectively. A median of 1000 inactive compound datapoints was calculated across targets and thresholds, with a median ratio of 0.4 active compounds to inactive compounds (see Additional file 1: Figure S1 for details). The dataset for putative inactives per target is available for download as zip files here: https://pidginv3.readthedocs.io/en/latest/install.html.

### Compound pre-processing

Compound structures were standardized using the IMI eTox molecular structure standardizer (https://github.com/flatkinson/standardiser), with settings to remove salts, waters, solvents, normalize charges, tautomerize (to the most favourable form) and to remove duplicates. RDKit [33] (Version 2019.03.4) was employed to remove structures without carbon, and to retain only compounds with atomic numbers between 21–32, 36–52, and greater than 53, and with a molecular weight between 100 and 1000 Da, to retain small organic molecule chemical space.

### Calculating uncertainty values for ChEMBL activity labels

Prior to the application of the PRF algorithm, the calculation of uncertainty in bioactivity labels was required. Since uncertainty originates from the hypothesis that bioactivity data extracted from public bioactivity databases have a degree of uncertainty, we introduced uncertainty into the labels. Thereby, labels were treated as probability distribution functions, rather than deterministic values by “injecting noise” in the following way. Bioactivity training data were converted into an uncertainty-based scale on a per-threshold basis ($pActivity^{T}$), across a range of arbitrary standard deviation (*σ*) thresholds ranging between 0.0 and 0.6, at increments of 0.2. By varying the standard deviation, $\sigma$, we evaluated model behaviour over a range of uncertainties.

For each bioactivity value ($pActivity$), we used the cumulative distribution function (cdf) of a normal distribution (Eq. 1) with a mean equal to the bioactivity threshold for each $pActivity^{T}$. More concretely, assuming only the mean and variance of activity values is known, the maximum entropy distribution to represent these values is a normal distribution [34]. One can set the mean and variance parameters of this distribution to a threshold value (e.g., 10 µM), and experimental error (e.g., $\sigma$ of 0.3) and compute the probability of activity values with the cdf. Each $pActivity$ value was converted to a y-label probability (*∆y*), a value representing the uncertainty in the measurement which was used for PRF training. We refer to this as the ‘ideal y-label’ or simply ‘y-ideal’, because it represents the ideal case, where experimental error is taken into account when training a target prediction model. For the calculation of *∆y*, the stats.norm.cdf() function was used from scipy [35] library in python as in Eq. 1:

1 $$\Delta y\left( {\vec{c}} \right) = \frac{1}{2}\left[ {1 + {\text{erf}}\left( {\frac{{\overrightarrow {{p_{Activity} }} - \overrightarrow {{p_{Threshold} }} }}{\sigma \sqrt 2 }} \right)} \right]$$

where ∆*y* were the y-label probabilities, $\vec{c} = (C_{1} , \ldots ,C_{n} )$ represented the compounds in the training set, $\overrightarrow {{p_{Threshold} }}$ described the pre-defined binding affinity thresholds for $\overrightarrow {{p_{Activity} }}$ (− log^10^) values, and $\sigma$ was the standard deviation defined in this work using arbitrary defined cut-offs (which could also be set as required to the deviation across replicates within or between experiments, screening platforms or activity unit aggregation methods).

**Fig. 2:**
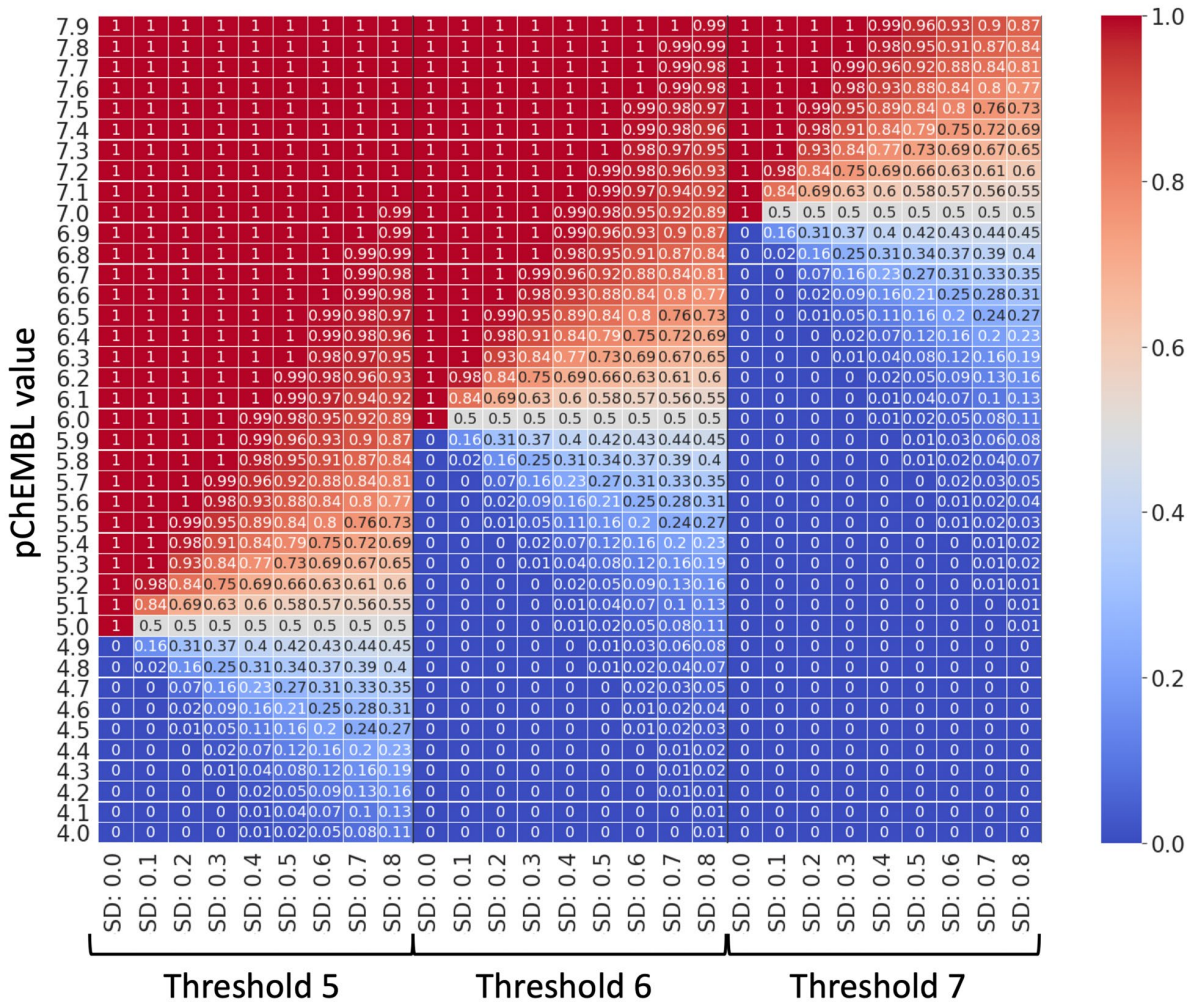
Schematic representation of how pChEMBL value is converted into the ideal y-label probability using cdf with different bioactivity thresholds and standard deviation (SD) values. The case when SD is 0 corresponds to traditional RF

Values of $\Delta y$ hence captured the likelihood that a given compound $C_{n}$ had binding affinity that falls within the boundary of the active class at the $p_{Threshold}$ given $p_{Activity}$ and given the assumption that most bioactivity data is homoscedastic (which is not always true in practice). Hence, a compound with a pChEMBL value of e.g., 5.1 (8 μM) was assigned a new *∆y* of ~ 0.63 for a pChEMBL activity threshold of 5.0 (10 μM) and a user-defined standard deviation $\sigma$ of 0.3 (Fig. 2), i.e., there is a 63% chance for that compound to belong to the active class given those parameters compared to traditional RF classifier which assumes that it is 100% active. This enabled representing the activity in a framework in-between the classification and regression architecture, with philosophical differences from either approach. Compared to classification, this approach enables better representation of factors increasing/decreasing inactivity. Conversely, one can utilize all data (even delimited/operand/censored data far from a cut-off) at the same time as taking into account the granularity around the cut-off, compared to a classical regression framework. Thereby, PRF combines characteristics from both classification and regression settings.

### Supplemental inactive data

**Fig. 3:**
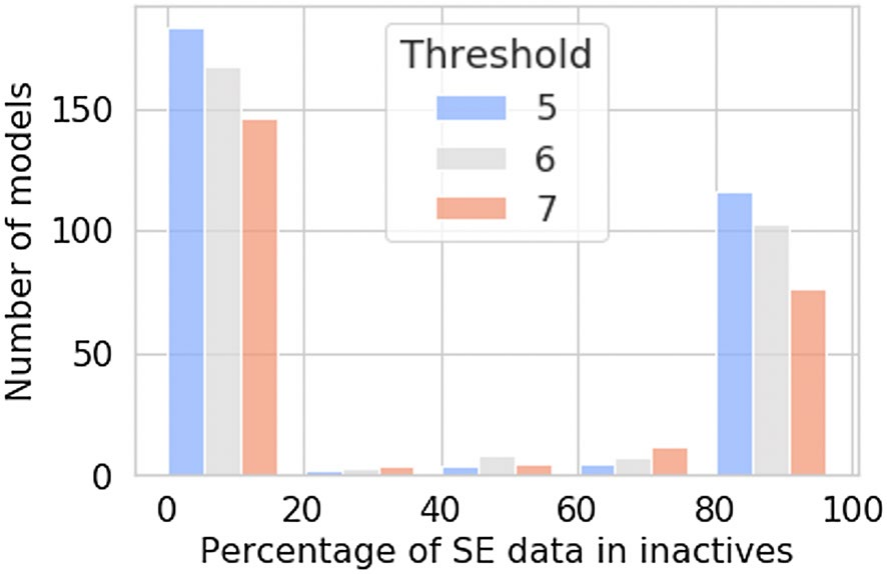
Percentage of sphere excluded inactive molecules included in the inactive molecule datasets of models across the three different bioactivity thresholds. Statistics show that the putative inactive compounds (calculated with sphere exclusion) account for up to 20% of the total inactive compounds for the majority of the targets that contain putative inactive compounds

In order to ensure sufficient chemical space of compounds not binding to targets (hence assigned a constant [$p_{Activity}$ = 0] across all test-train standard deviations) an inactive dataset of compounds from PubChem was used as published in Mervin, et al. [36] and available at https://github.com/lhm30/PIDGINv3. These supplemental inactive compounds were randomly sampled from PubChem with a Tanimoto coefficient fingerprint similarity to actives lower than 0.4 to obtain the desired number of compounds, which could reasonably be assumed to be inactive against a given target. The dataset included 38,902,310 inactive labelled compound annotations across the full complement of targets. For these inactive datapoints, *∆y* remained constant across test-train *σ* thresholds (i.e., only bioactivity data points from ChEMBL were assigned ∆y probabilities greater than zero). In more detail, out of a total of 557 models trained (e.g., with a pXC_50_ threshold equal to 5), 310 models (~ 56%) included at least 1 SE datapoint in the inactive set of compounds and the percentage of SE data included in the inactive data of the 310 models is shown in Fig. 3. As we observe, 183 models (33% of total models) were trained with a small number of SE data of about 20% of the total inactive compounds and 116 models (21% of total models) were trained with a high number of SE data points (more than 80% SE data in the inactive compound set).

### Machine learning modelling and benchmarking

#### Random Forest

The Probabilistic Random Forest (PRF) is a modification to the original RF algorithm; hence we first outline the RF concept followed by the modifications to enable uncertainty estimation via the PRF.

RF is an ensemble method using a number of decision trees during training. Each decision tree is described via a tree-like graph relating the relationships between (chemical) features and target (activity) variables in a series of conjoined conditions arranged in a top-to-bottom “tree-like” structure. For binary classification, trees are constructed via nodes searching for the ‘best split’; the combinations of features and thresholds providing the best separation between classes [37]. Gini impurity, the probability that a randomly selected object (compound) will be misclassified if assigned a randomly selected label (i.e., active or inactive), is frequently employed for this purpose.. Let $P_{n,A}$ and $P_{n,B}$ denote the fractions of objects of classes A and B within the group in the node n (class probabilities), hence the Gini impurity $G_{n}$ is:

2 $$G_{n} = 1 - \left( {P_{n,A}^{2} + P_{n,B}^{2} } \right)$$

The algorithm iterates over features and thresholds dividing training data “left” or “right” corresponding to objects left or right of the threshold, respectively. The splitting threshold resulting in the minimal combined impurity of the groups is defined as:

3 $$G_{n, right} x f_{n,right} + G_{n, left} x f_{n,left}$$

where G_right_, G_left_ in node n are Gini impurities and f_right_, f_left_ are the fractions of objects in each group. This iteration process over features and thresholds is repeated recursively (so long as groups have a lower combined impurity compared to the impurity of the node) until ending in a terminal node (which assigns probabilities according to the distribution of compounds in the classes). Novel predictions are propagated through the tree with predictions assigned via the largest fraction of samples in terminal nodes.

Individual decision trees are prone to overfitting since they are engineered so as to perfectly fit all samples in the training data set. To combat this, a RF is a set of many decision trees, with randomness introduced via: (1) randomly sampled subsets of the full dataset, and (2) random subsets of the features in each node of the trees. Aggregation across the randomised decision trees reduces the tendency of overfitting. An unlabelled object is propagated through the trees in the forest, and the predicted class probability for an input sample computed as the mean predicted fraction of samples of the same class in the terminal nodes across the trees. Both (a) the fraction of the trees voting for a predicted class and (b) deviation of the fraction of samples in the terminal nodes across the forest can serve as certainty measures for predictions.

#### Probabilistic Random Forest

RFs receive a sample of observed random pairs of random variables, $\left( {x_{1} ,y_{1} } \right), \ldots ,\left( {x_{n} ,y_{n} } \right)$ describing the relation: $h:X \to Y$ used to predict $y$ for a given value of $x$. On the other hand, the PRF receives $\left( {x_{1} ,y_{1} ,\Delta x_{1} ,\Delta y_{1} } \right), \ldots ,\left( {x_{n} ,y_{n} ,\Delta x_{n} ,\Delta y_{n} } \right)$, where ∆x and ∆y represent uncertainty in features and labels, respectively. Naturally, the focus of this work is concerned with (activity) label uncertainties, and (chemical) feature uncertainties are not specified.

To account for uncertainty, the PRF treats labels as normal distributions, rather than deterministic values. Labels become probability mass functions (PMFs) where each object has a label assigned to it with some probability and the relationship between RF and PRF follow naturally from this concept, since the PRF converges toward a RF when there are low or no (zero) uncertainties in *∆y* (see Fig. 2). Another difference between the two algorithms is that randomness of a RF is induced epistemically (i.e., from the model itself) by training different decision trees on randomly selected subgroups of the data and by using random subsets of features in each node of each decision tree. On the other hand, PRF introduces randomness allosterically; since it is not drawn from a defined distribution, but rather the underlying uncertainty (experimental deviation) relevant for classification. Label uncertainties propagate through the splitting criterion during the construction of the tree. Similar to a standard tree, nodes are split left and right, such that resulting subsets are more homogeneous than the set in the parent node. A cost function for minimization is used for this purpose since the transition from *y* to *∆y* means that labels now become random variables. Instead of calculating the fraction of objects in node, n, the expectancy value ($\pi_{i} \left( n \right))$ is calculated:

4 $$\begin{gathered} P_{n,A} \to \overline{P}_{n,A} = \frac{{\mathop \sum \nolimits_{i \in n} \pi_{i} \left( \eta \right) x p_{i,A} }}{{\mathop \sum \nolimits_{i \in n} \pi_{i} \left( \eta \right)}} \hfill \\ P_{n,B} \to \overline{P}_{n,B} = \frac{{\mathop \sum \nolimits_{i \in n} \pi_{i} \left( \eta \right) x p_{i,B} }}{{\mathop \sum \nolimits_{i \in n} \pi_{i} \left( \eta \right)}} \hfill \\ \end{gathered}$$

Hence, Gini impurity is transformed to:

5 $$G_{n} \to \overline{G}_{n} = 1 - \left( {\overline{P}_{n,A}^{2} + \overline{P}_{n,B}^{2} } \right)$$

The cost function (weighted average of the modified impurities of the two nodes) is then:

6 $$\overline{G}_{{\left( {n,r} \right)}} x \frac{{\mathop \sum \nolimits_{{i \in \left( {n,r} \right)}} \pi_{i} \left( {\eta ,r} \right) }}{{\mathop \sum \nolimits_{i \in n} \pi_{i} \left( \eta \right)}} + \overline{G}_{{\left( {n,l} \right)}} x \frac{{\mathop \sum \nolimits_{{i \in \left( {n,l} \right)}} \pi_{i} \left( {\eta ,l} \right) }}{{\mathop \sum \nolimits_{i \in n} \pi_{i} \left( \eta \right)}}$$

The modified propagation scheme and cost functions are the two major conceptual changes separating PRFs and RFs. After training, the PRF classifies new objects which is identical for both training and prediction. Once an object reaches a terminal node the class probability can be used to provide the prediction as in the classical RF, since each object reaches all the terminal nodes a probability. Hence, all the predictions given by all the terminal nodes should be taken into account to obtain the prediction of the tree, which is given by the following equations:

7 $$Pr_{A} \to \mathop \sum \limits_{terminal nodes} \pi \left( n \right) x \overline{P}_{n,A}$$

8 $$Pr_{B} \to \mathop \sum \limits_{terminal nodes} \pi \left( n \right) x \overline{P}_{n,B}$$

### Computational details

The PRF implementation in Reis, Baron and Shahaf [30] was employed for this work as provided via https://github.com/ireis/PRF. The algorithm was fit with the RDKit fingerprints and the corresponding ∆y labels on a per standard deviation (*σ*) basis, with a lower propagation probability limit (“keep_proba”) of 0.05, to ensure that a given object did not propagate to branches with a low probability (reducing runtime without impairing performance). The output of the PRF was recorded as the number of probabilistic decision trees in the forest predicting the label. The RF was implemented using the RandomForestClassifer function from Scikit-Learn.

Two different metrics were used to compare the PRF and RF prediction probabilities. The first metric is the error margin as described in Eq. 9:

9 $$Error\;margin = \left[ {\begin{array}{*{20}c} {\left( {ideal \; ylabel - {\varvec{RF}}\;probabilities} \right) - } \\ {\left( {ideal \;ylabel - {\varvec{PRF}}\;probabilities} \right)} \\ \end{array} } \right]$$

In addition to the error margin, when two scores are compared (y-probability from 1. RF and 2. PRF) rather than comparing only the absolute values, it is also possible to compare the scores relative to each other. This is achieved by calculating the relative increase toward the potential optimum (i.e., the ideal y-label) as shown in Eq. 10:

10 $$Relative\;score = \frac{{\left| {\left| {error\;margin\;RF} \right| - \left| {error\;margin\;PRF} \right|} \right|}}{{error\;margin \left( {worst\;performing\;classifier} \right)}} \times 100$$

The rationale behind this calculation is that for a metric with an ideal y-label e.g., equal to 0.65 a difference between RF and PRF y-probabilities from 0.75 to 0.70 is more meaningful than a difference from 0.85 to 0.80. In terms of relative score, the latter and the former difference in y-probabilities correspond to 50% and 25% change respectively.

### Evaluation of Sphere Exclusion effect on the fraction of improved models by PRF

The effect of including sphere excluded putative inactives on the error margins by Probabilistic RF was evaluated. In this comparison, we selected (a) targets that did not contain any putative inactives (models without SE data) and (b) targets that 80% of their inactive datapoints were putative inactives (models with SE data) across the three different bioactivity thresholds and different emulated test-train standard deviations. We calculated the error margin between the two algorithms (as described in the section above) separately for models without SE data and models with SE data across different standard deviations. As a result, we derived two error margin distributions and sought to compare their means to understand if there is a statistically significant difference. Firstly, a Kolmogorov Smirnov (KS) test in scipy (scipy.stats.kstest) was applied to confirm if the data in error margin distributions are normally distributed. Next, an unpaired t-test (scipy.stats.ttest_ind, with ‘equal_var’ parameter equal to False) was applied to statistically compare the distributions.

## Results & discussion

### ChEMBL experimental variability

**Fig. 4:**
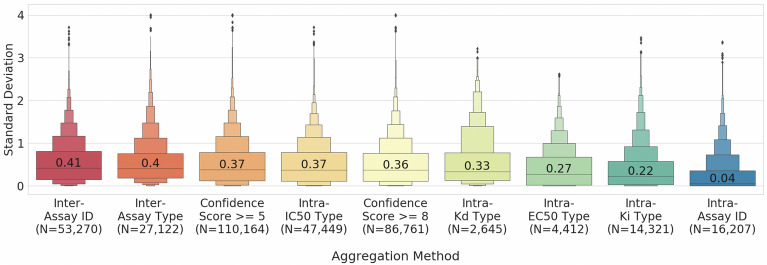
Standard deviation of replicate affinity measurements (IC_50_/EC_50_/K_i_/K_d_) across different aggregation types. Standard deviations range between a median of ~ 0.04 to 0.41 depending on the method of aggregation used for cross-comparison. The median values are shown in each box

We first evaluated the standard deviation across various aggregation schemes for the bioactivity data in ChEMBL 27, as outlined in Additional file 2: Table S1, to better understand the influence of different approaches toward aggregation as a product of the observed standard deviation between replicate measurements for the same compound-protein target pair. Results from this analysis are presented in Fig. 4. It can be seen that there is a standard deviation between 0.22–0.41 depending on method of bioactivity data aggregation between the different grouping schemes. For example, two replicates with pChEMBL values of 5.6 and 6.3 have a standard deviation of 0.40 and if we train a model with a threshold equal to 6, then there is a degree of uncertainty whether we should consider the compound as active or inactive. As expected, the smallest median deviation in experimental values of 0.04 was observed within the same experiment (replicate) for “intra-assay” aggregation, when compound-target pair replicates were compared within the same experiment. On the other hand, we observed a high standard deviation (0.41) in experimental values across different assay ids and the main reason is that different assay protocols were being used. Though there is an effort to better document and report experimental details regarding assays [38], significant variability was observed between measurements taken in different labs even when assay conditions appear to be the same. In addition, as previously outlined in the work of Kalliokoski et al. [16], aggregation across IC_50_ values was problematic and produced one of the highest median standard deviations of 0.37 for the “Intra IC_50_ type” bin. This is because IC_50_ values are assay-specific and comparable only under certain conditions, which also illustrates the danger of pooling IC_50_ values from different experiments, as is frequently done in the literature (mostly due to lack of alternatives).

From our observation we conclude that decisions should be taken when aggregating data from databases because of trade-off between increasing data set size versus increasing the discrepancies between the assay technologies and reported activity types (K_i_ vs IC_50_). Therefore, one needs to vary the standard deviation depending on the data that is being modelled and how stringent the aggregation function that has been employed.

### Probabilistic random forest (PRF) performance

**Fig. 5:**
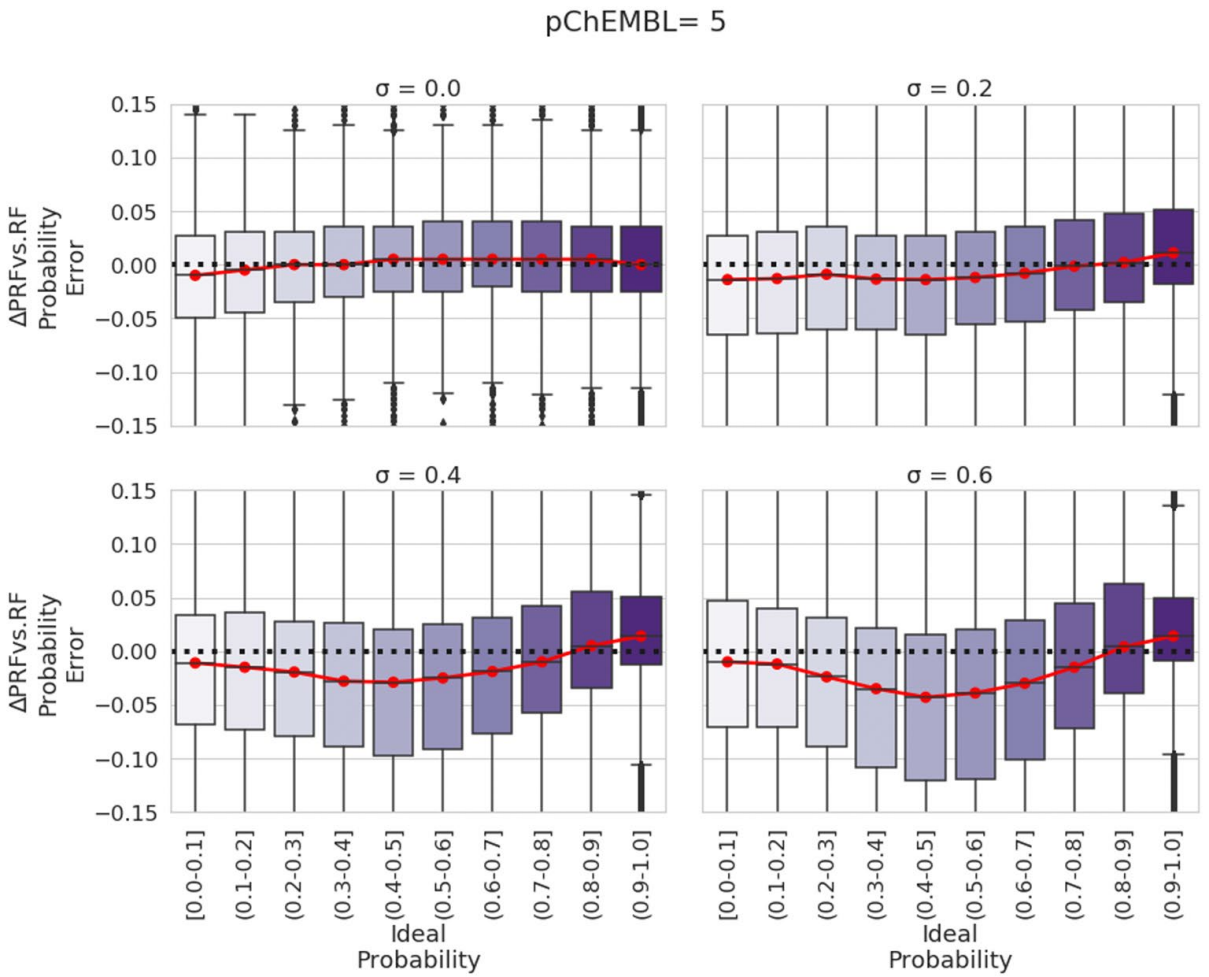
Ideal probabilities as a function of the delta of PRF versus RF error margins across emulated train-test standard deviations. Overall, results shown here for a threshold of pChEMBL value of 5 (0.1 µM) highlight the most optimal PRF probability estimates were observed in cases when standard deviation in the test set most closely resembled that in the training set. It can also be seen that the largest benefit in terms of error margin for the PRF (lower values on the y-axis) are observed toward the midpoint of the ideal ∆y scale, particularly for higher training set standard deviations. This is when the original RF weights the marginal cases equivalent in distinguishing between activity classes. The same observation was observed for pChEMBL thresholds of 6 and 7, as shown in Additional file 1: Figure S3, S4, respectively

In a first step toward benchmarking the PRF, we first evaluated which method (RF or PRF) performs better by taking into account uncertainty around the bioactivity threshold. The difference of performance between PRF and RF was defined as the difference between RF error margin and PRF error margin. Error margin was the difference of each classifier’s predicted probability to the ‘ideal’ y-label probability calculated with the cumulative distribution function (which takes into account both bioactivity threshold and a range of pre-defined values of *σ* for both test and train sets). Results of this analysis for a pChEMBL cut-off of 5 (0.1 μM) are outlined in Fig. 5 (complete analysis of pXC_50_ 5, 6 and 7 with different combination of SD in train and test set are included in Additional file 1: Figure S2–S4, respectively).

Figure 5 shows that PRF outperformed RF when there was a degree of uncertainty in the data (i.e., a σ greater or equal to 0.2). For example, when the σ = 0, the median error margin between the two algorithms was close to 0 (− 0.010 to 0.005) across all y-ideal probabilities. However, we observed that as the standard deviation in the data increased, the absolute error margin between the two algorithms was increasing too. When e.g., σ = 0.4 and σ = 0.6 the median error margin ranged from − 0.029 to 0.005 and from − 0.039 to 0.004 respectively. Therefore, these results indicated that when σ of training data is 0, there were no substantial differences in the predictions between algorithms and this was not true as the standard deviation increased. This observation is in agreement with previous benchmarking of PRF in a different type of noisy data (astronomical data) [30] and the difference in classification accuracy between the two algorithms (RF and PRF) increased with increasing noise level and complexity.

Moreover, Fig. 5 highlights areas in the y-ideal probability ranges, where PRF outperformed RF. For example, when there was an uncertainty in the data and σ was equal to 0.2, 0.4 and 0.6, PRF outperformed RF with an average absolute error margin equal to 0.011, 0.024 and 0.037 for y-ideal probability ranges of 0.4–0.6. However, when y-ideal probability ranged from 0.7 to 1, the absolute error margin between the two algorithms was smaller and equal to 0.005, 0.009 and 0.011 for σ equal to 0.2, 0.4 and 0.6 respectively. A similar trend was observed when the y-ideal probability ranged from 0.0 to 0.3 and the absolute error margin was equal to 0.012, − 0.015, 0.015. Therefore, PRF showed a highest absolute error margin and thus was outperforming RF for the y-ideal probabilities closer to midpoint. Therefore, the PRF exhibited the largest benefit over the RF (defined as the lowest delta between PRF error and Scikit-Learn RF error) toward the midpoint of the probability scale, for marginal cases on the binary threshold boundary. This is because the original RF weights the marginal cases as equivalent in distinguishing between activity classes. In this case the PRF classifier was able to better model the granularity around the activity threshold cut-off, as in a regression.

The findings reported above are specific to an analysis using the Scikit-Learn implementation RF. In order to check that the above findings are robust and not due to differences between packages, a similar analysis was conducted emulating a classical RF (i.e., when the binary labels are supplied rather than the probabilities) via the PRF package, as described in the methods. A high overall R^2^ correlation between Scikit-Learn RF and the PRF (*σ* = 0) ranging between ~ 0.97–0.98 across the standard deviation test sets was observed (as presented in Additional file 1: Figure S5), hence the returned predictions from both RF approaches were overall comparable and the findings presented in this study are robust between packages.

We next investigated the significances of differences between the RF and PRF modelling uncertainties, based on the differences between output and expected values (y-ideal probability). To evaluate this, we applied the relative score calculation as described in the methods, to identify the percentage improvement for each algorithm across different emulated train-test standard deviations and different ranges of ideal y-label. As shown in Table 2, PRF showed the greatest percentage improvement (~ 17%) when SD of train and test set ranged from 0.4 to 0.6 and when the ideal y-label ranged from 0.4 to 0.6 and thus the data were close to the bioactivity threshold. Thus, the improvement of correct class assignments showed that PRF has an advantage compared to RF when there was a degree of uncertainty in the data and additionally PRF performed better for values toward the midpoint of the probability scale as also shown across algorithm error margins in Fig. 5.

**Table 2:** Average percentage improvement between RF and PRF probabilities in relation to ideal y-label values across different emulated train-test standard deviations (SDs) when pChEMBL threshold equals 5

Standard deviation in train and test set	y-ideal range (N)	Better- performing Algorithm	% improvement
SD-train: 0.0–0.4 & SD-test: 0.0–0.4	0.0–0.2 (183,255)	PRF	4.79
	0.2–0.4 (79,890)	PRF	3.83
	0.4–0.6 (124,505)	PRF	10.8
	0.6–0.8 (166,210)	PRF	5.76
	0.8–1.0 (1,007,685)	RF	6.57
SD-train: 0.4–0.8 & SD-test:0.4–0.8	0.0–0.2 (152,835)	PRF	0.27
	0.2–0.4 (194,300)	PRF	9.27
	0.4–0.6 (339,495)	PRF	16.89
	0.6–0.8 (592,575)	PRF	11.04
	0.8–1.0 (5,624,495)	RF	9.59

Overall, we have shown in this section that PRFs were able to capture the experimental/aggregational variability in ChEMBL. We have shown that the maximum achievable accuracy of PRF models was more closely related to the true reproducibility across the experimental data (in this case when aggregated across experiments and measurement data types). In comparison, the baseline RF (when *σ* = 0) yielded a reported performance smaller than the experimental uncertainty, which indicated cases of overfitting and/or over-confidence. Therefore, PRF is an algorithm that should be considered as an alternative to RF when we have a priori knowledge that our training data are noisy.

### Effect of Sphere Exclusion, dataset imbalance and model set size

**Fig. 6:**
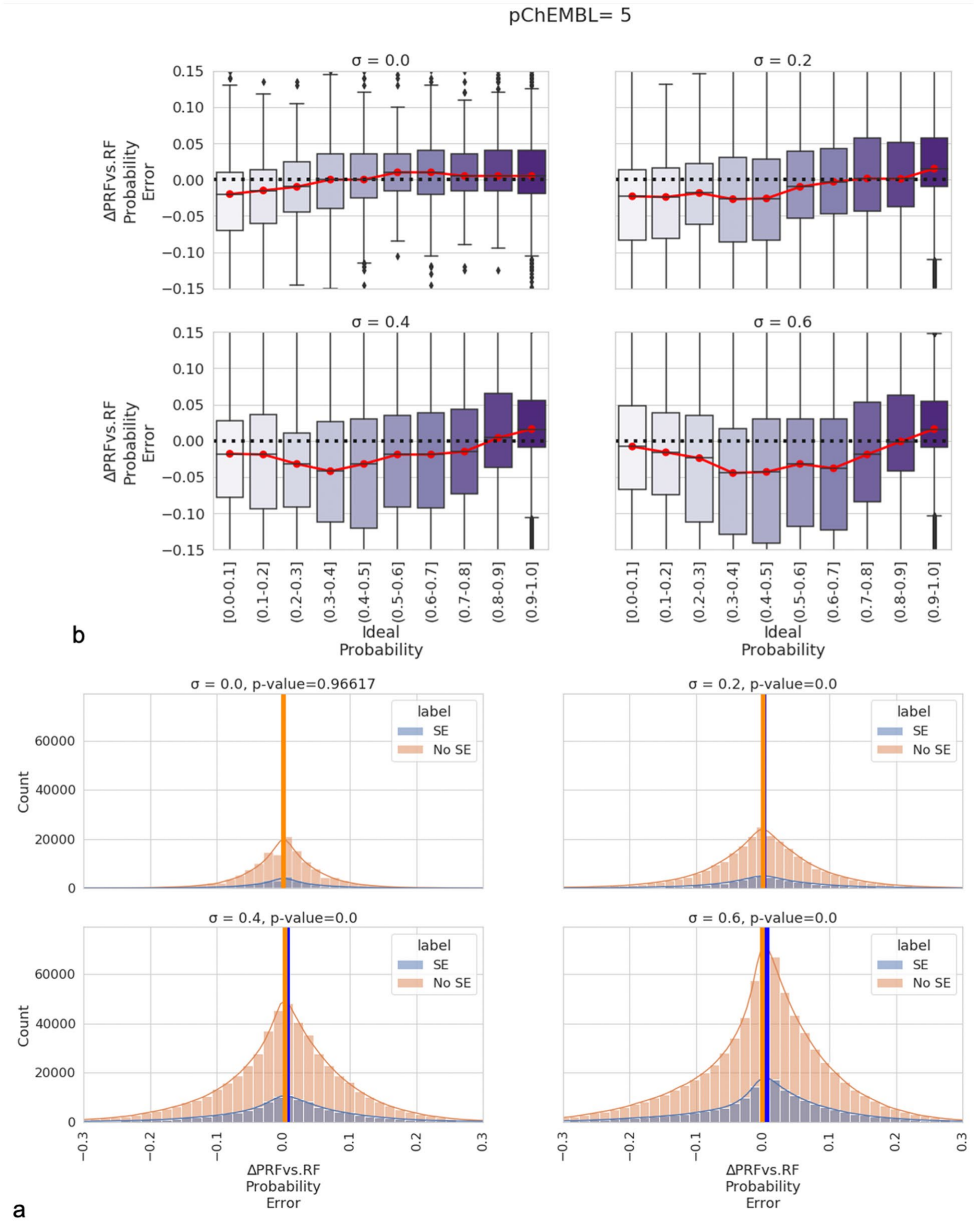
**a** Ideal y-probabilities as a function of the delta of PRF versus RF error margins across emulated train-test standard deviations. Overall, results shown here for a threshold of pChEMBL value of 5 (10 µM) highlight the most optimal PRF probability estimates were observed in cases when standard deviation in the test set most closely resembled that in the training set. It can also be seen that the largest benefit in terms of error margin for the PRF (lower values on the y-axis) are observed toward the midpoint of the ideal ∆y scale, particularly for higher training set standard deviations. This is when the original RF weights the marginal cases equivalent in distinguishing between activity classes. **b** Effect of Sphere Exclusion (SE) on the error margin between models with and without SE data across different emulated test-train standard deviations. Overall results show that there is no clear advantage of including or excluding SE data when there is no SD in the data. When SD is greater or equal to 0.2, there is a statistically significant difference and hence the inclusion of SE data reduces performance of PRF

Previous studies link Sphere Exclusion (SE) with inflated model performance and poor model calibration (due to the artificial requirement for putative non-binding molecules to be dissimilar to their active counterparts [39, 40]). Conversely, experimentally confirmed inactive compounds are likely to be more skeletally similar to actives and this trend blurs the algorithm’s decision boundary between the active and inactive classes. Hence, we next sought to evaluate whether the presence of SE inactives influenced PRF performance by comparing the fraction of targets improved by PRF with the classical RF, for models with/without putative inactives. We first explored the error margin between PRF and RF for target protein models that included a high number of putative inactives in Fig. 6a (detailed comparison shown in Additional file 1: Figure S6) and for targets that did not include any putative inactives (Additional file 1: Figure S7). Overall, results showed that the PRF exhibited the largest benefit over the RF toward the midpoint of the probability scale, for marginal cases on the binary threshold boundary and when there was a degree of uncertainty in train and test set (otherwise for low SD PRF converged to classic RF). These observations are in agreement with the previous observations in Fig. 5, where we evaluated the error margin for all the models and thus the addition of putative inactive compounds did not affect the performance of PRF compared to RF.

In addition, we explored the effect of including sphere excluded putative inactive compounds on the error margins between the two algorithms separately for models without SE data and models with SE data across different standard deviations. By applying a Kolmogorov Smirnov (KS) test, the data in error margin distributions were normally distributed and therefore we applied an unpaired t-test to compare them. The error margin distributions and the result of unpaired t-test are shown in Fig. 6b. Overall, results showed that there was no statistically significant difference between models with and without SE data when SD was equal to 0. However, as the SD increased (0.2, 0.4 and 0.6), there was a statistically significant difference between the error margins of the models with and without SE data with p-values less than 0.05. The addition of SE data reduced the difference between PRF, and RF compared to models without SE data. The rationale behind this observation could be that for the putative inactives, we cannot assign a pXC_50_ value and thus evaluate their uncertainty and therefore they are considered as inactives with a low uncertainty and i.e., far from the bioactivity threshold. Therefore, a large number of putative inactives could be problematic when combined with PRF but on the other hand their inclusion can enlarge the models’ applicability Domain.

We next investigated how significant are the differences between RF and PRF in terms of how close they are to the real value (y-ideal probability). To this end, we applied the relative score (Eq. 10) calculation to identify the percentage improvement for each algorithm across different training conditions (standard deviation in train and test set) and different ranges of y-ideal range for the targets that included at least 1 SE datapoint in the inactive dataset as shown in Table 3. The main observation is that PRF showed the greatest percentage improvement 11.58% and 14.68% when SD of train and test set ranged from 0.0 to 0.4 and 0.4 to 0.6 respectively and when the ideal y-label ranged from 0.4 to 0.6 and thus the datapoints were located close to the bioactivity threshold. On the other hand, RF showed a ~ 12% improvement when ideal y-label ranged from 0.8 to 1.0 and therefore RF worked better for datapoints that were assigned as actives with a high confidence. Therefore, we observed that the inclusion of SE data did not affect the percentage of improvement in different SDs and y-ideal probability changes.

**Table 3:** Average percentage improvement between RF and PRF probabilities in relation to ideal y-label values across different emulated train-test standard deviations (SDs) when pChEMBL threshold equals 5

Standard Deviation in train and test set	y-ideal range (N)	Better-performing Algorithm	% improvement	Average Percentage of SE data
SD-train: 0.0–0.4 & SD-test: 0.0–0.4	0.0–0.2 (104,345)	PRF	6.63	38.66
	0.2–0.4 (42,075)	PRF	5.19	34.03
	0.4–0.6 (63,520)	PRF	6.42	36.74
	0.6–0.8 (86,27)	PRF	3.19	36.01
	0.8–1.0 (530,080)	RF	6.96	31.57
SD-train: 0.4–0.6 & SD-test:0.4–0.6	0.0–0.2 (92,720)	PRF	0.23	42.68
	0.2–0.4 (106,755)	PRF	11.65	35.82
	0.4–0.6 (173,070)	PRF	16.76	36.08
	0.6–0.8 (314,270)	PRF	11.60	33.52
	0.8–1.0 (3,022,800)	RF	9.48	29.99

In a final analysis, we sought to evaluate the influence of dataset size on the performance difference of PRF versus traditional RF models. Our correlation analysis, (presented in Additional file 1: Figure S8) showed no discernible correlation between PRF versus RF performance and training size, since no significant Pearson correlation exists across the four arbitrary standard deviations *(σ*) evaluated (Pearson *r* values ranged between − 0.22 to − 0.03). We can hence conclude that PRF can be used, regardless of dataset size, for cases when experimental uncertainty is large and where values are distributed around the classification threshold.

### Case study: PRF improves PDK1 model performance

After taking into account the learnings from the previous analyses, we concluded that PRF exhibited the largest benefit over the RF toward the midpoint of the probability scale, i.e. for marginal cases on the binary threshold boundary. Therefore, we selected one particular target to highlight how PRF can be useful to predict compounds near the bioactivity threshold with higher confidence compared to classic RF.

**Fig. 7:**
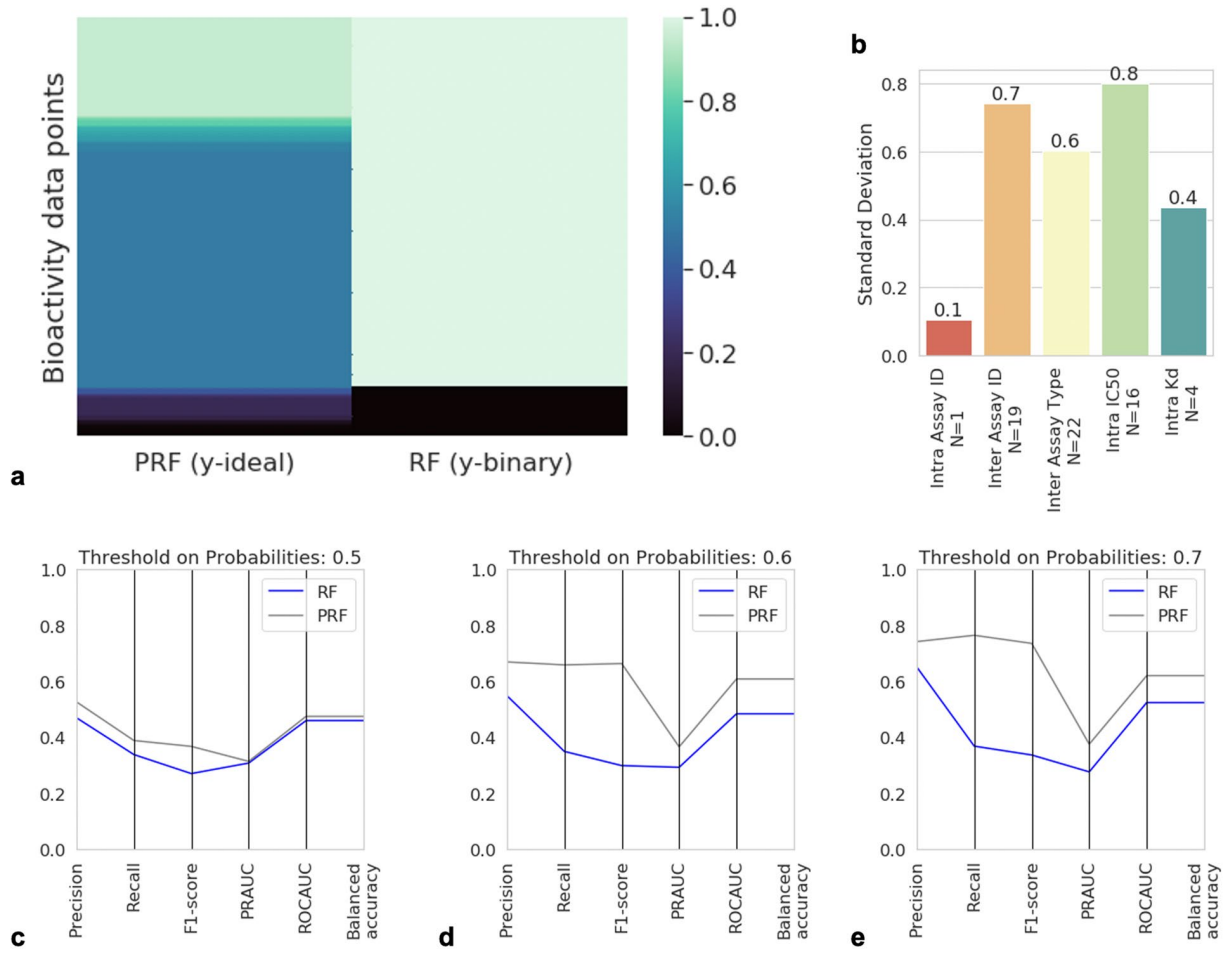
**a** Distribution of the y-ideal label versus binary y-labels for values close to bioactivity threshold. **b** Experimental error in ChEMBL for [Pyruvate dehydrogenase (acetyl-transferring)] kinase isozyme. We observe that the error is high when data are derived from different assay IDs and IC_50_ measurements. **c**–**e** Performance of the PRF versus RF classifier using different evaluation metrics and different thresholds on algorithms probabilities and y-ideal labels

The protein target selected for this analysis was Pyruvate dehydrogenase kinase isozyme 1, encoded by the PDK1 gene, which has been investigated as a potential drug target for breast cancer, due to its essential role in regulating cell migration [41]. This particular target was chosen due to the large proportion of reported activity data measured close to the bioactivity threshold, (i.e., ~ 60% of the training labels for PRF ranged between 0.3–0.6), as shown in Fig. 7a. This behaviour can be contrasted to the distribution of binary labels for the classical RF, where the majority of labels (1000 compounds) were assigned (0) for the “non-binding” class. We first performed the replicate analysis (analogous to the one presented in “ChEMBL experimental variability” section). One replicate from the same assay showed a low standard deviation of 0.1 whilst the majority of other replicates (across assays and measurement types) showed higher deviations around ~ 0.3, as outlined in Fig. 7b. Hence, replicate aggregation is shown for this target to introduce uncertainty into the bioactivity labels in accordance with the global analysis previously outlined. Finally, using different thresholds on the raw probabilities (0.5, 0.6, 0.7) returned by PRF and RF, we observed that PRF outperformed the traditional RF and maintained a higher performance compared to RF even when we used a higher threshold on probabilities (Fig. 7c–e). This illustrates the benefit of taking experimental uncertainty into account using the PRF classifier, as opposed to a RF classifier, for protein targets where much of the data is located around the decision boundary on a concrete dataset.

## Summary

In conclusion, the aim of this analysis was to investigate the performance of Probabilistic Random Forest (PRF) as a method able to take into account experimental errors, which are usually a neglected aspect of model generation. By evaluating the current experimental error in ChEMBL v27, we identified that it is very similar to those reported in previous versions of ChEMBL v14. The highest standard deviation in values for the same ligand-target interaction pairs observed for values derived from different assay types and the smallest deviation in experimental values is observed within the same assay id. By applying PRF in target prediction and comparing it to RF we identified cases where PRF outperforms RF and vice versa. Therefore, the choice should be based on (a) training data quality and (b) the area of data distribution (i.e., whether they are close to the classification threshold). Firstly, regarding the training data quality we observed that PRF showed a lower error compared to RF when there is a degree of uncertainty in training set (i.e., SD ≥ 0.2). For lower SD in the data (when the uncertainties are set to or close to zero), the PRF converges to the original RF algorithm. When the standard deviation of training set is 0, there are no substantial differences in the prediction of the test set regardless of the standard deviation assigned in the test data. Secondly, PRF exhibits the largest benefit over the RF toward the midpoint of the probability scale, i.e. for marginal cases on the binary threshold boundary. In addition, we evaluated whether the addition of sphere excluded inactives affects PRF performance compared to RF and SE data did not affect the observations obtained from the comparison of RF vs PRF. Therefore, we conclude that PRF can be useful for target prediction and is not affected by the presence of SE data. Based on our observations, we particularly recommend using PRF for classification in cases where experimental uncertainty is large, and where values are distributed around the classification threshold.

## Supplementary Information

**Additional file 1: Figure S1.** a) Number of Active and Inactive compounds across the 559 models and across the three different pXC_50_ Thresholds (5, 6, and 7). **Figure S2.** Ideal probabilities as a function of the delta of PRF versus RF error margins across emulated train-test standard deviations. **Figure S3.** Ideal probabilities as a function of the delta of PRF versus RF error margins across emulated train-test standard deviations. **Figure S4.** Ideal probabilities as a function of the delta of PRF versus RF error margins across emulated train-test standard deviations. **Figure S5.** Comparison between RF scikit-learn implementation and PRF (when *σ* = 0). **Figure S6.** Ideal probabilities as a function of the delta of PRF versus RF error margins across emulated train-test standard deviations for models trained with a min of 80% putative inactives. **Figure S7.** Ideal probabilities as a function of the delta of PRF versus RF error margins across emulated train-test standard deviations for models trained without putative inactives. **Figure S8.** Correlation analysis of model sizes (when pChEMBL threshold is 5 [10 µM]) as a function of PRF improvement.

**Additional file 2: Table S1.** Standard deviation of replicate affinity measurements (IC_50_/EC_50_/K_i_/K_d_) across different aggregation methods.

## Data Availability

The ChEMBL dataset used to train the models are available at the GitHub repository: https://github.com/BenderGroup/PRF. The inactive compound dataset from extracted from PubChem is available at the GitHub repository: https://github.com/BenderGroup/PIDGINv4. The code used to train and evaluate the PRF models are available at the GitHub: https://github.com/BenderGroup/PRF.
