# Supplementary material for: Probabilistic Random Forest improves bioactivity predictions close to the classification threshold by taking into account experimental uncertainty
Source: J Cheminform. 2021 Aug 19;13:62. doi: 10.1186/s13321-021-00539-7 (PMC8375213; doi:10.1186/s13321-021-00539-7)
Supplement: Supplementary file 1 — Additional file 1: Figure S1. a) Number of Active and Inactive compounds across the 559 models and across the three different pXC50 Thresholds (5, 6, and 7). Figure S2. Ideal probabilities as a function of the delta of PRF versus RF error margins across emulated train-test standard deviations. Figure S3. Ideal probabilities as a function of the delta of PRF versus RF error margins across emulated train-test standard deviations. Figure S4. Ideal probabilities as a function of the delta of PRF versus RF error margins across emulated train-test standard deviations. Figure S5. Comparison between RF scikit-learn implementation and PRF (when σ = 0). Figure S6. Ideal probabilities as a function of the delta of PRF versus RF error margins across emulated train-test standard deviations for models trained with a min of 80% putative inactives. Figure S7. Ideal probabilities as a function of the delta of PRF versus RF error margins across emulated train-test standard deviations for models trained without putative inactives. Figure S8. Correlation analysis of model sizes (when pChEMBL threshold is 5 [10 µM]) as a function of PRF improvement. [file 13321_2021_539_MOESM1_ESM.docx]

**Probabilistic Random Forest improves bioactivity predictions close to the classification threshold by taking into account experimental uncertainty**

Lewis H. Mervin^1§*^, Maria-Anna Trapotsi^2*^, Avid M. Afzal^3^, Ian P. Barrett^3^, Andreas Bender^2^ and Ola Engkvist^4,5^

**Additional Material**

**Additional Figures**

| a)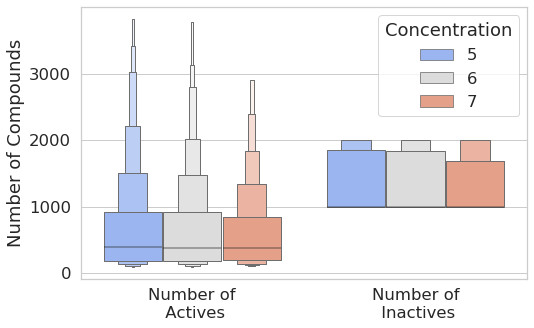 | | |
| --- | --- | --- |
| b) pXC_50_ Threshold = 5 | c) pXC_50_ Threshold = 6 | d) pXC_50_ Threshold = 7 |
| 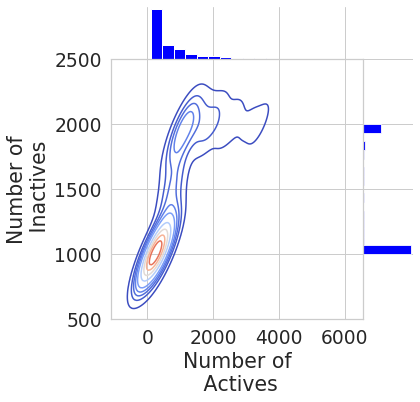 | 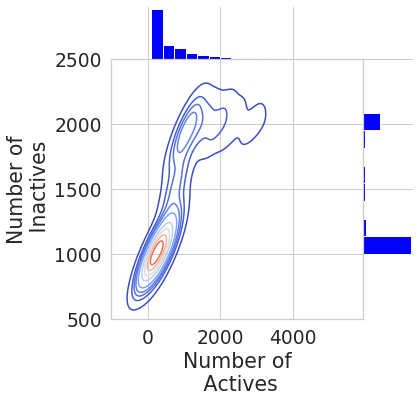 | 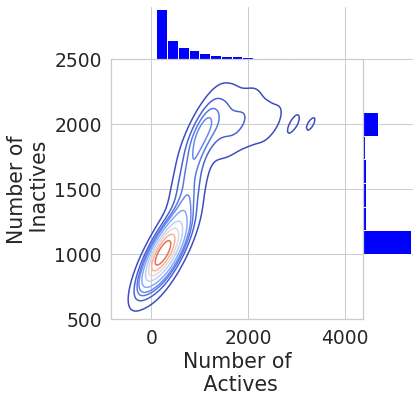 |

**Figure S1:** a) Number of Active and Inactive compounds across the 559 models and across the three different pXC_50_ Thresholds (5, 6, and 7). Number of Actives vs the Number of Inactives per model across the three different pXC_50_ Thresholds: b) 6, c) 6 and d) 7.

**
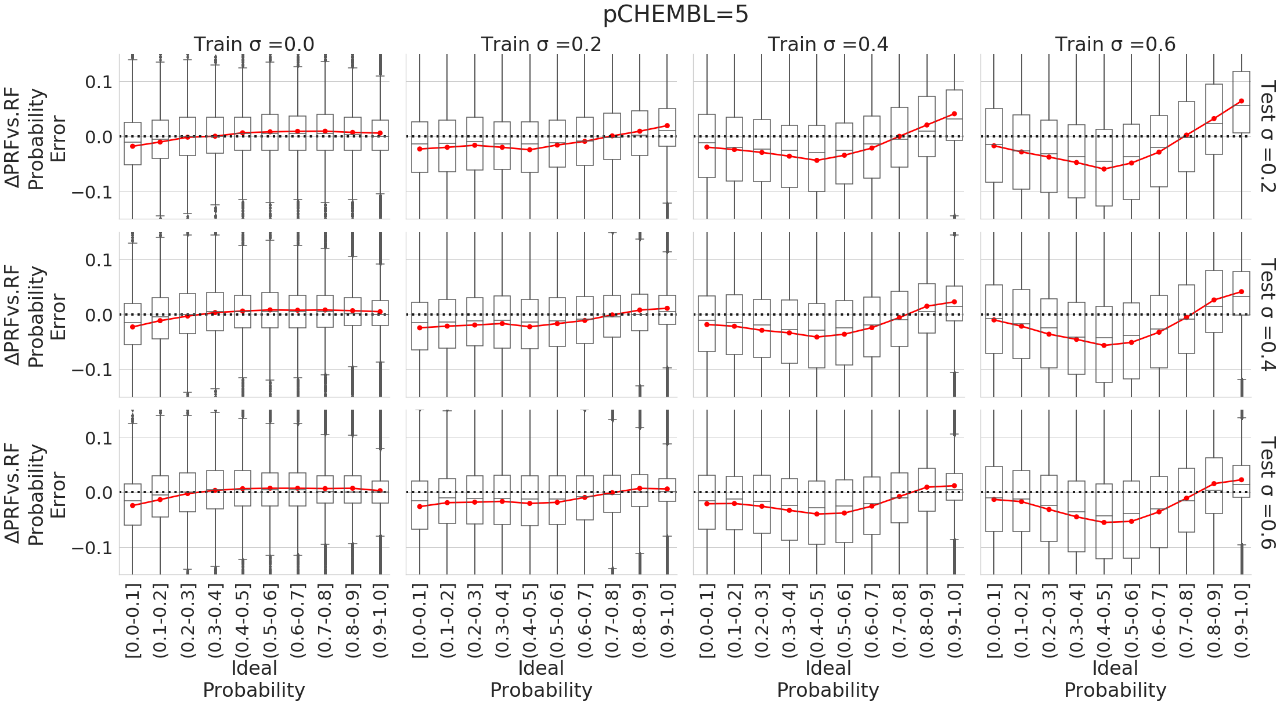
Figure S2:** Ideal probabilities as a function of the delta of PRF versus RF error margins across emulated train-test standard deviations. Overall, results shown here for a threshold of pChEMBL value of 5 highlight the most optimal PRF probability estimates were observed in cases when standard deviation in the test set most closely resembled that in the training set. It can also be seen that the largest benefit in terms of error margin for the PRF (lower values on the y-axis) were observed toward the midpoint of the ideal ∆y scale, particularly for higher training set standard deviations. This is when the original RF weights the marginal cases equivalent in distinguishing between activity classes.


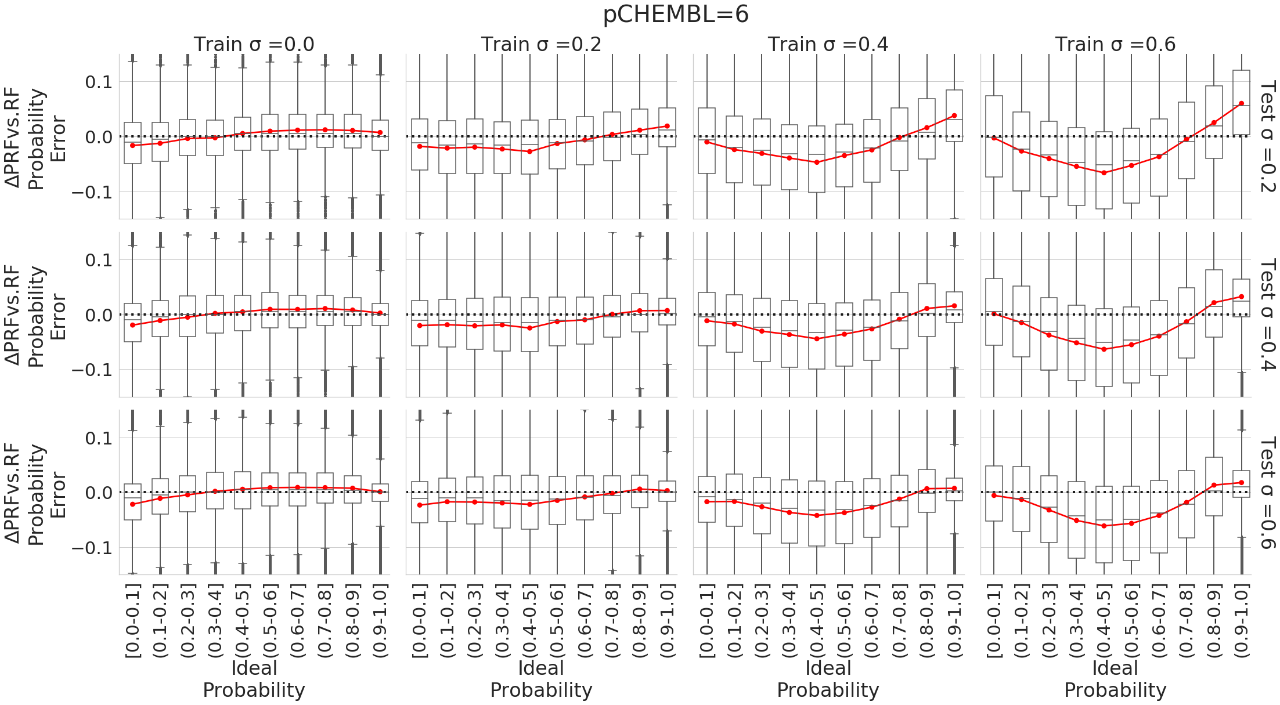
**Figure S3**: Ideal probabilities as a function of the delta of PRF versus RF error margins across emulated train-test standard deviations. Overall, results shown here for a threshold of pChEMBL value of 6 highlight the most optimal PRF probability estimates were observed in cases when standard deviation in the test set most closely resembled that in the training set. It can also be seen that the largest benefit in terms of error margin for the PRF (lower values on the y-axis) were observed toward the midpoint of the ideal ∆y scale, particularly for higher training set standard deviations. This is when the original RF weights the marginal cases equivalent in distinguishing between activity classes.


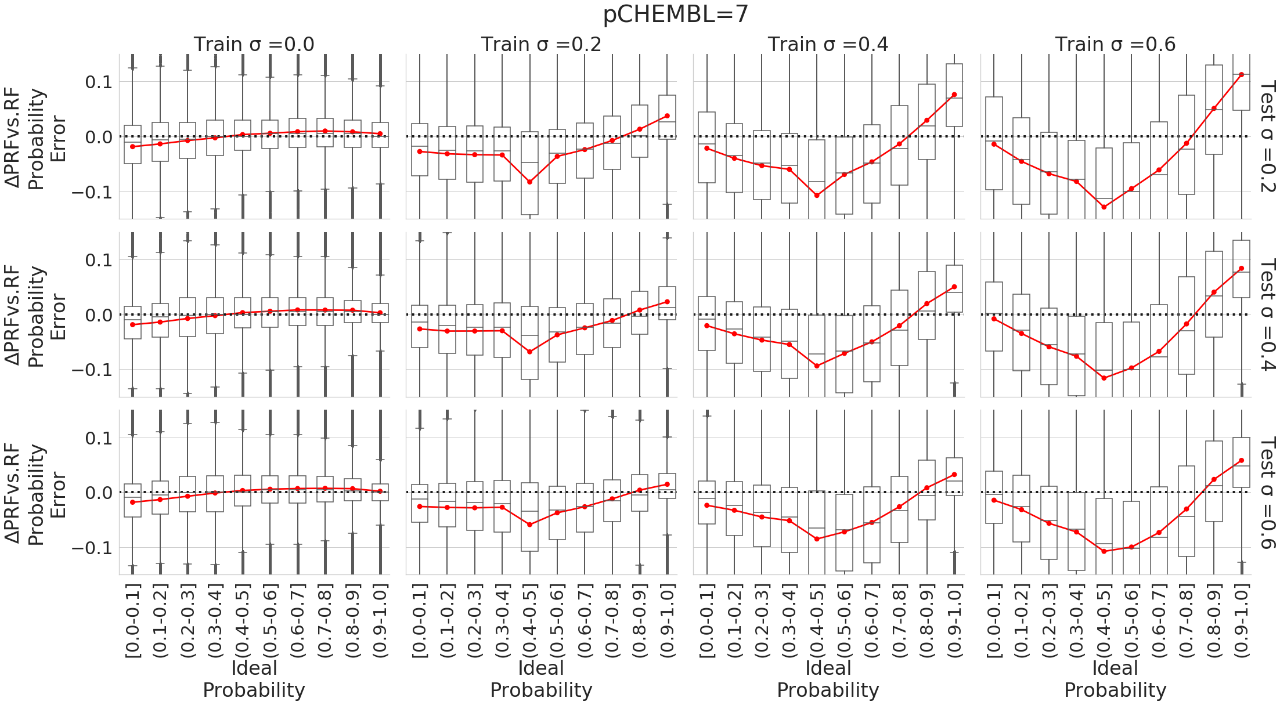


**Figure S4**: Ideal probabilities as a function of the delta of PRF versus RF error margins across emulated train-test standard deviations. Overall, results shown here for a threshold of pChEMBL value of 7 highlighted the most optimal PRF probability estimates were observed in cases when standard deviation in the test set most closely resembled that in the training set. It can also be seen that the largest benefit in terms of error margin for the PRF (lower values on the y-axis) are observed toward the midpoint of the ideal ∆y scale, particularly for higher training set standard deviations. This is when the original RF weights the marginal cases equivalent in distinguishing between activity classes.


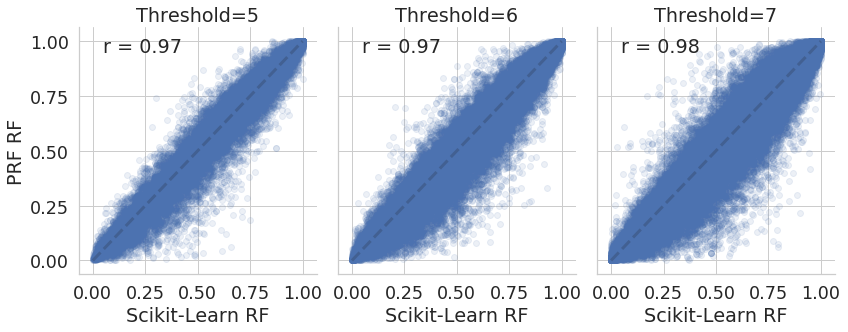


**Figure S5:** Comparison between RF scikit-learn implementation and PRF (when *𝜎* = 0). There was a high overall R^2^ correlation between Scikit-Learn RF and the PRF (*𝜎* = 0) ranging between ~0.97-0.98 across the standard deviation test sets, hence the returned predictions from both RF approaches are overall comparable.


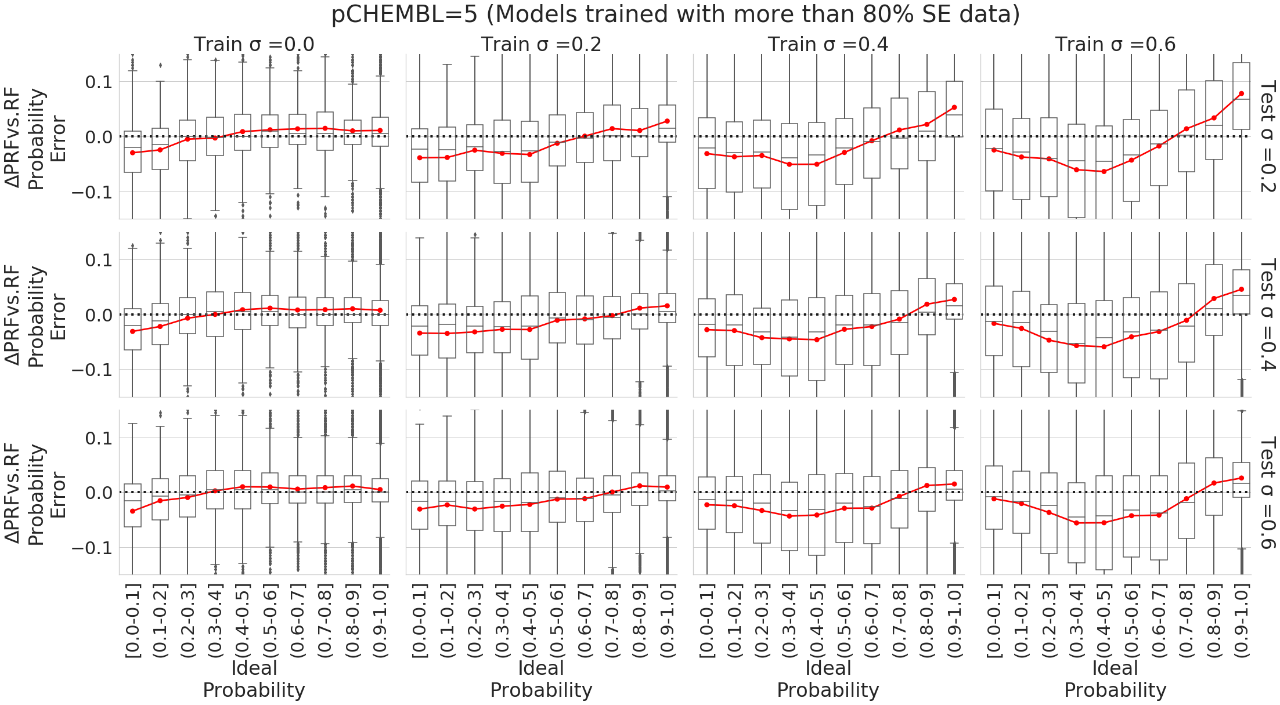


**Figure S6**: Ideal probabilities as a function of the delta of PRF versus RF error margins across emulated train-test standard deviations for models trained with a min of 80% putative inactives. Overall, results shown here for a threshold of pChEMBL value of 5 (10 µM) highlight the most optimal PRF probability estimates were observed in cases when standard deviation in the test set most closely resembled that in the training set. It can also be seen that the largest benefit in terms of error margin for the PRF (lower values on the y-axis) are observed toward the midpoint of the ideal ∆y scale, particularly for higher training set standard deviations. This is when the original RF weights the marginal cases equivalent in distinguishing between activity classes.


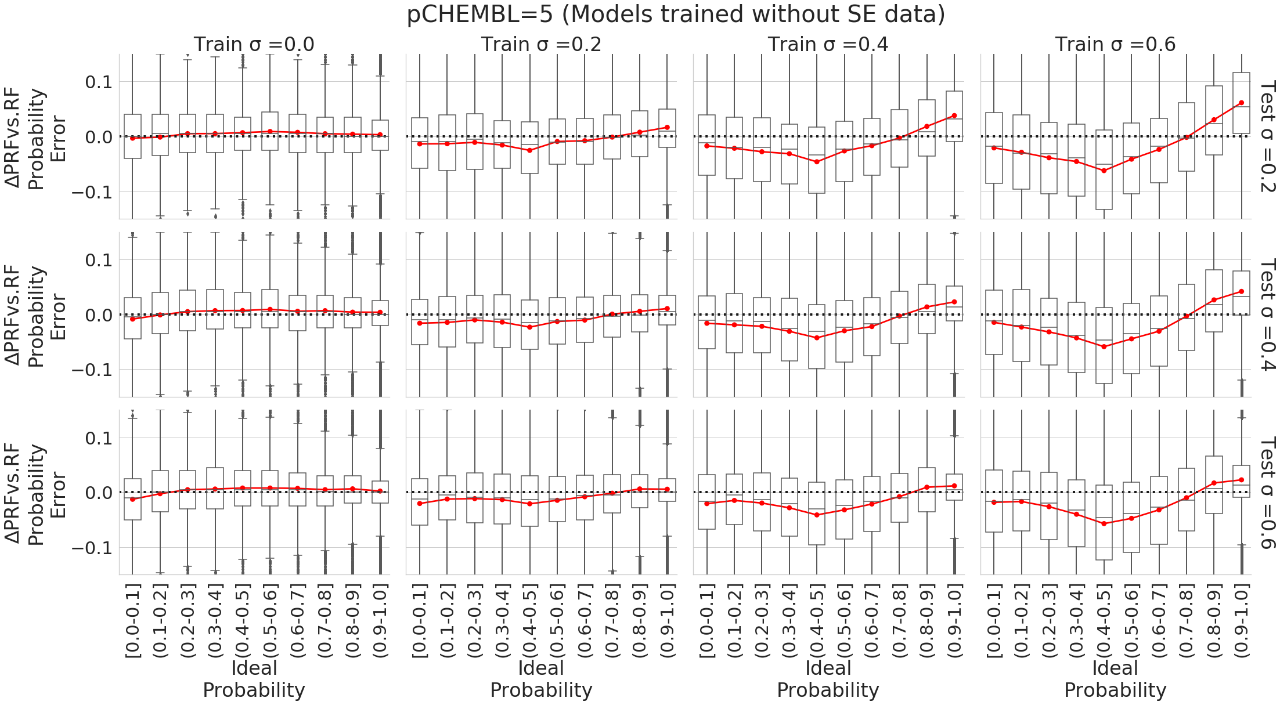


**Figure S7:** Ideal probabilities as a function of the delta of PRF versus RF error margins across emulated train-test standard deviations for models trained without putative inactives. Overall, results shown here for a threshold of pChEMBL value of 5 (10 µM) highlight the most optimal PRF probability estimates were observed in cases when standard deviation in the test set most closely resembled that in the training set. It can also be seen that the largest benefit in terms of error margin for the PRF (lower values on the y-axis) are observed toward the midpoint of the ideal ∆y scale, particularly for higher training set standard deviations. This is when the original RF weights the marginal cases equivalent in distinguishing between activity classes.


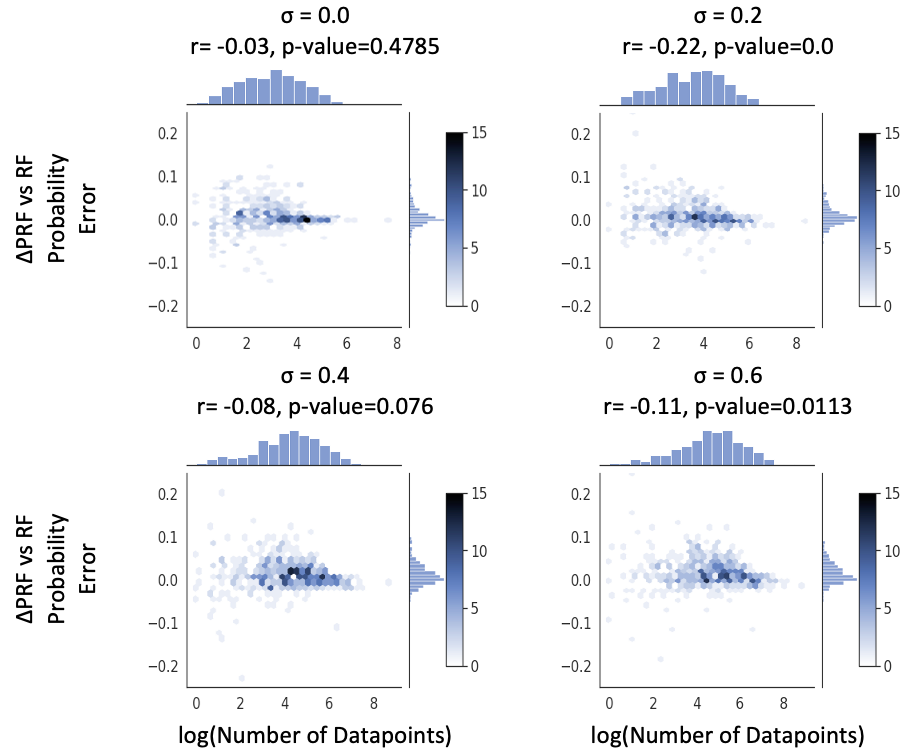


**Figure S8**: Correlation analysis of model sizes (when pChEMBL threshold is 5 [10 µM]) as a function of PRF improvement. Overall, model dataset size was shown to have no discernible effect on the improvement of PRF versus the baseline RF, since no significant Pearson correlation exists across the four arbitrary standard deviations *(𝜎*) evaluated. Increased density is represented via the blue hex marker intensity.
