# Supplementary material for: Probabilistic Random Forest improves bioactivity predictions close to the classification threshold by taking into account experimental uncertainty
Source: J Cheminform. 2021 Aug 19;13:62. doi: 10.1186/s13321-021-00539-7 (PMC8375213; doi:10.1186/s13321-021-00539-7)
Supplement: Supplementary file 2 — Additional file 2: Table S1. Standard deviation of replicate affinity measurements (IC50/EC50/Ki/Kd) across different aggregation methods. [file 13321_2021_539_MOESM2_ESM.docx]

Table S1: **Standard deviation of replicate affinity measurements (IC_50_/EC_50_/K_i_/K_d_) across different aggregation methods.** The mean, median and variance of standard deviation values across replicates are presented.

| Aggregation Method | Number of replicate measurements | Mean | Median | Variance |
| --- | --- | --- | --- | --- |
| Inter-Assay ID | 53,270 | 0.55 | 0.41 | 0.26 |
| Inter Assay Type | 27,122 | 0.55 | 0.40 | 0.27 |
| Confidence score >= 5 | 110,164 | 0.53 | 0.37 | 0.27 |
| Confidence score >= 8 | 86,761 | 0.51 | 0.37 | 0.26 |
| Intra-IC_50_ Type | 47,449 | 0.51 | 0.36 | 0.25 |
| Intra-K_d_ Type | 2,645 | 0.56 | 0.33 | 0.37 |
| Intra- EC_50_ Type | 4,412 | 0.42 | 0.27 | 0.23 |
| Intra-K_i_ Type | 14,321 | 0.40 | 0.22 | 0.25 |
| Intra-Assay ID | 16,207 | 0.26 | 0.04 | 0.18 |
